# What Factors Hindered the Access to Essential Anticancer Medicine in Public Hospitals for the Local Population in Hubei Province, China

**DOI:** 10.3389/fphar.2021.734637

**Published:** 2021-09-21

**Authors:** Chaoyi Chen, Zhanchun Feng, Yufeng Ding, Ziqi Yan, Jia Wang, Ruoxi Wang, Da Feng

**Affiliations:** ^1^ School of Medicine and Health Management, Tongji Medical College of Huazhong University of Science and Technology, Wuhan, China; ^2^ Tongji Hospital Affiliated to Tongji Medical College of Huazhong University of Science and Technology, Wuhan, China; ^3^ School of Pharmacy, Tongji Medical College of Huazhong University of Science and Technology, Wuhan, China

**Keywords:** essential anti-cancer medicine, availability, affordability, price, cancer

## Abstract

**Background:** *Cancer* poses a serious threat to one’s health, which caused significant economic burden on the family and society. Poor availability and affordability resulted in some essential medicines failing to meet the basic health needs of this group of patients. The objective of this study was to evaluate the availability, prices and affordability of 32 anticancer essential medicines in Hubei Province, China.

**Methods:** Data on the availability and price related information of 32 essential anticancer medicines in the capital and five other cities of Hubei Province were collected. A total of 28 hospitals were sampled, which included 13 tertiary hospitals and 15 secondary hospitals. We used the standard methods developed by the World Health Organization and Health Action International to compare the differences in drug price, availability and affordability between secondary hospitals and tertiary hospitals.

**Results:** Overall, the availability of medicine was higher in tertiary hospitals. The average availability of originator brand (OBs) was 13.70% (tertiary hospitals) VS 6.67% (secondary hospitals), and lowest-priced generic (LPGs) was 62.83% (tertiary hospitals) VS 42.92% (secondary hospitals). The MPR value of most sampled medicines in secondary hospitals were less than 1. In contrast, the MPR of Cytarabine (17.15), Oxaliplatin (12.73) were significantly higher than the international reference price. The top three OBs’ total expenses for 30-days treatment were Irinotecan, Oxaliplatin, Bicalutamide. Further, their affordability was relative low, as the costs for one course using these medicines were much higher than 20% of the minimum family monthly income.

**Conclusion:** Though the “Zero Mark-Up” and “Centralized procurement policy of anti-tumor drugs” policies have been implemented in China, the availability issue yet to be addressed. High price and low affordability were the major barriers to the access of essential anticancer medicines. Measures should be taken to provide sufficient, available and affordable medicines to patients in need.

## Introduction

*Cancer* has become a serious health problem, which contributed to severe burden of disease and even death (Zhu et al., 2019). A total of 9.6 million deaths due to cancer diseases were reported worldwide in 2018 (Erratum, 2020). China accounts for 21.0% of the worldwide new cancer cases, and total of 22.96 million Chinese residents died of cancer (Shaoqing, 2019). At present, anticancer medicine is a core component of treatment, but barriers to receiving cancer therapy such as high price of anticancer medicines and poor availability were yet to be solved (Smith et al., 2014). These problems may be related to medical information asymmetry and the distribution of responsibilities between the drug purchasing sector and the patients who bear the cost restrict competition in the market (Hasan et al., 2019).

Essential medicines are the indispensable drugs that satisfy the priority health care needs of the population. Meanwhile, they are selected with due regard to rational use and equitable access on their efficacy, safety and comparative cost-effectiveness (Beran et al., 2018). To better meet the necessary treatment needs, the Chinese government attaches great importance to the development and implementation of the Essential Medicine List (EML) (Khanal et al., 2019). In 2018, China re-adjusted the national EML, and increased the growth rate of oncology drugs by 35%. In terms of cancer treatment, the 2018 edition of the “National Essential Medicine List” added five kinds of non-targeted drugs, including Ifosfamide, Gemcitabine, Pingyangmycin, Capecitabine, and Letrozole (Dong et al., 2020). The supplementary medicines can greatly alleviate the burden of cancer treatment.

In addition, the price of 14 anticancer drugs fell by an average of 14.95% in China, as the National Medical Security Bureau issued centralized drug purchase measures and accelerated the pace of medical insurance drug-price negotiation (Chinese, Government, 2018; Kaiyue et al., 2021). Despite medical insurance policy system was improved, dozens of targeted anticancer drugs have been included in category B (local medical expense settlement institutions could bear approximate 55–70% of the patients’ cost of anticancer drug) (Kunhe et al., 2017), due to the unbalanced distribution of medical resources and the per capita financing guarantee level of China’s medical insurance is still relatively weak, the total amount of medical insurance controlled by provinces and hospitals has limited funds allocated for health care, as a result, many cancer drugs still have high treatment costs even after medical insurance promotion policies are implemented (Yf).

Recently, most of domestic studies on essential medicines focused on antibacterial drugs and medications for chronic diseases (Wu et al., 2018; Dong et al., 2020; Liang et al., 2020). Their study can reflect the common problems in the availability and affordability of essential drugs in China, however, the question regarding whether it is the same case for anticancer still awaits further investigation. Therefore, we investigated the price, availability and affordability of anticancer drugs in 28 public hospitals in Hubei Province, in order to explore the factors influencing the shortage of medicines and rapidly increasing health expenditure, and accordingly, propose corresponding strategies to the government and pharmaceutical management institutes.

## Methods

A cross sectional survey was conducted from November 2018 to January 2019. Data were collected on medicine price, availability and affordability information from tertiary hospitals and secondary hospitals in six cities of Hubei Province following WHO/HAI standardized approach and Health Action International (HAI) (Khuluza and Heide, 2017).

### Study Design/Sampling

#### Area Selection

We selected six cities as survey regions in Hubei Province by referring to the Gross Domestic Product (GDP) in 2018, according to which, we selected three cities in high-income (Wuhan, Yichang, Xiangyang) and low-income (Huanggang, Xianning, Suizhou) areas, respectively (China YearBooks, 2019). Hospitals were selected using a multistage clustered approach. Firstly, we chose the best tertiary hospital as the survey anchor (it ranked the first in this area) in each city (for instance, Tongji affiliated hospital of Tongji medical college was selected in Wuhan city). After identifying the survey anchor, we selected at least one secondary hospital pharmacy, which was closest to the anchor hospital. Since Baidu maps was used to select secondary hospital pharmacies from a list of all facilities within a 3-h drive from each survey anchor hospital. But if back-up outlet was less than 50% of the medicines on the Medicine Price Data Collection form, then we would select another secondary hospital pharmacy in the sample list. Last, a total of 13 tertiary hospitals and 15 secondary hospitals in the public sector were selected from six cities in Hubei Province. As anticancer drugs belong to the category of key controlled drugs in China, hence, they are mainly available in secondary and tertiary hospitals of China. So we don’t include retail pharmacies in our investigation.

### Medicine Selection

A total of 32 medicines were included in this survey, referring to 2017 WHO Essential Medicine List (WEML) and 2018 National Essential Medicine List (NEML). Considering the common acute and medication conditions in tertiary and secondary hospitals of Hubei Province, we divided the selected medicines into two parts according to the available data collected from the sample hospitals. Firstly, the intersection area of anticancer drugs were from the WHO Essential Medicine List and National Essential Medicine List, which included 20 core essential medicines. Then, additional 12 sample drugs were also included in this survey list as supplementary according to oncologist’s recommendation and the actual needs of local hospitals. For each medicine in the survey, data were collected for two medicine types: the originator brand (OBs), and the lowest-priced generic (LPGs) equivalent found at each hospital.

### Data Collection

With the aid of skilled pharmacy present, trained investigators visited the pharmacy department of the sampled tertiary and secondary hospitals. They collected information regarding the cost and availability using a standardized data collection form. At the end of each day, two students would enter the data into a designed MS Excel Workbook provided as a part of the WHO/HAI methodology (Eden et al., 2019; Saeed et al., 2019). The items included in the standardized form were as follows: basic information of the facility (hospital name, hospital level, survey date), information of the medicine (medicine in stock in the hospital on the day of data collection, yes or no), dosage, strength, medicine type (OB/LPG), and the retail price at the time of survey (Table 1).

**TABLE 1:** List of 32 anticancer medicines surveyed in the Hubei Province.

Name	Strength	Dosage Form	Volume	WHO EML	NEML
Calcium Folinate	100 mg/10 ml	VIAL	1	no	yes
Capecitabine	500 mg	TAB-CAP	12	yes	yes
Carboplatin	100 mg/10 ml	VIAL	1	no	no
Ciclosporin	25 mg	TAB-CAP	50	yes	yes
Cisplatin	20 mg	VIAL	1	no	yes
Cyclophosphamide	200 mg	VIAL	1	no	yes
Cytarabine	100 mg	VIAL	1	yes	yes
Daunorubicin	10 mg	VIAL	1	no	no
Docetaxel Trihydrate	20 mg/ml	VIAL	1	yes	no
Doxorubicin	10 mg	VIAL	1	yes	yes
Etoposide	20 mg/ml	VIAL	1	yes	no
Fluorouracil	0.25 g/10 ml	VIAL	1	no	yes
Gemcitabine	200 mg	VIAL	1	yes	yes
Ifosfamide	500 mg	VIAL	1	yes	yes
Imatinib	100 mg	TAB-CAP	60	yes	yes
Irinotecan	40 mg/2 ml	VIAL	1	yes	no
Mercaptopurine	50 mg	TAB-CAP	50	yes	yes
Mesna	0.4 g/4 ml	AMPOULE	1	yes	yes
Methotrexate Sodium	100 mg/ml	VIAL	1	no	no
Methylprednisolone	500 mg	VIAL	1	no	no
Mycophenolate Mofetil	250 mg	TAB-CAP	40	no	yes
Oxaliplatin	50 mg	VIAL	1	yes	yes
Paclitaxel	30 mg/5 ml	VIAL	1	yes	yes
Tamoxifen Citrate	10 mg	TAB-CAP	60	yes	yes
Vincristine	1 mg	VIAL	1	yes	yes
Letrozole	2.5 mg	TAB-CAP	10	no	yes
Gefitinib	250 mg	TAB-CAP	10	no	yes
Bicalutamide	50 mg	TAB-CAP	28	yes	no
Hydroxycarbamide	500 mg	TAB-CAP	100	yes	yes
Vinorelbine	10 mg/ml	VIAL	1	yes	no
Tacrolimus	1 mg	TAB-CAP	10	no	no
Ondansetron	4 mg	TAB-CAP	10	yes	yes

### Patient and Public Involvement

It was not possible or appropriate to involve patients or the public in the design, data collection, reporting or dissemination plans of this analysis.

### Assessment of Availability

The availability of anticancer medicines was reported as the percentage (%) of medicine outlets in which the information was documented by surveyed facilities on the day of data collection. Availability was classified as 4°; **Absent**: 0 of facilities, suggested that we could not find these medicines in the facility; **Low**: < 50%, these medicines were rarely purchased; **Fairly high**: 50–80%, these medicines were found in several facilities; **High**: > 80%, most institutions sold these medicines. In addition, mean percentage availability of OBs and LPGs were calculated for the investigations (Zhu et al., 2019).

### Price Assessment

In order to make standard comparisons, price was evaluated by median price ratio (MPR) according to WHO/HAI approach, which is the ratio of median price of individual medicine obtained during the survey (Dong et al., 2020). Moreover, we used Management Sciences for Health (MSH 2015) International Drug Price Indicator Guide as the source of reference prices. MPR was calculated using the formula given below: Median Price Ratio (MPR) = Median local unit price/International reference unit price. It can provide a more intuitive data presentation for drug price monitoring: when the value of MPR is less than 1, it indicates that the price of the drug under investigation is lower than the international average standard. In other words, the price control is efficient. The value of MPR between 1 and 2 indicates that the drug price is acceptable; if MPR>2, this means that the drug price level is high and needs to be contained (Guan et al., 2018).

### Affordability

In general, the total cost of the unit treatment for chronic diseases with the standard dose of the drug is equivalent to 1 month’s working days (daily wages) of the lowest paid non-technical government employee that enables him/her to purchase the standard course of cancer treatment. However, the duration of anticancer therapy may be longer. Moreover, the price of many anticancer drugs is too expensive, which causes heavy economic burden for cancer patients. Thus, with the aim to precisely assess the affordability, we used the approach proposed by Khatib and Sarwar (Khatib et al., 2015; Sarwar et al., 2018): if the total expenditure of medicine in 30 days accounted for 20% or less of the minimum family monthly income, it was regarded as affordable (Eden et al., 2019). The calculation formula was:

$$Affordability=\frac{\mathrm{Total cost of drugs in 30 days}}{\mathrm{20\% of the minimum monthly household income}}$$

## Results

We calculated the availability of originator brands (OBs) and lowest priced generics (LPGs) across the 32 anticancer medicines. In this survey, 92.43% OB was unavailable overall, and 10 OBs of 32 medicines were not available in tertiary hospitals nor secondary hospitals. The top three percentage availability of OBs in tertiary hospitals included Bicalutamide (47.62%), Methylprednisolone (38.10%) and gefitinib (28.57%). In tertiary hospitals, the average availability of OBs was 13.70%. In contrast, the mean availability of OBs was 6.67% in secondary hospitals (Table 2).

**TABLE 2:** Availability of anticancer medicines in Tertiary and Secondary hospitals.

Medicine name	OBs(%)		LPGs (%)		Total (%)	
	Tertiary hospital	Secondary hospital	Tertiary hospital	Secondary hospital	OBs	LPGs
Calcium Folinate	0.00	0.00	47.62	60.00	0.00	53.81
Capecitabine	23.81	40.00	52.38	66.67	31.90	59.52
Carboplatin	0.00	0.00	52.38	33.33	0.00	42.86
Ciclosporin	23.08	6.67	38.10	13.33	10.48	25.71
Cisplatin	0.00	0.00	42.86	53.33	0.00	48.10
Cyclophosphamide	15.38	0.00	52.38	93.33	4.76	72.86
Cytarabine	7.69	0.00	28.57	13.33	2.38	20.95
Daunorubicin	0.00	0.00	28.57	26.67	0.00	27.62
Docetaxel Trihydrate	7.69	0.00	47.62	73.33	2.38	60.48
Doxorubicin	0.00	0.00	23.81	33.33	0.00	28.57
Etoposide	0.00	0.00	57.14	80.00	0.00	68.57
Fluorouracil	7.69	0.00	33.33	46.67	2.38	40.00
Gemcitabine	7.69	0.00	61.90	73.33	2.38	67.62
Ifosfamide	23.08	0.00	33.33	13.33	7.14	23.33
Imatinib	7.69	6.67	38.10	20.00	5.71	29.05
Irinotecan	7.69	6.67	47.62	46.67	5.71	47.14
Mercaptopurine	0.00	0.00	4.76	0.00	0.00	2.38
Mesna	7.69	0.00	38.10	20.00	2.38	29.05
Methotrexate Sodium	0.00	0.00	52.38	66.67	0.00	59.52
Methylprednisolone	38.10	40.00	9.52	26.67	39.05	18.10
Mycophenolate Mofetil	7.69	13.33	28.57	13.33	9.05	20.95
Oxaliplatin	15.38	6.67	61.90	93.33	8.10	77.62
Paclitaxel	23.08	0.00	42.86	40.00	7.14	41.43
Tamoxifen Citrate	0.00	0.00	52.38	80.00	0.00	66.19
Vincristine	0.00	6.67	28.57	46.67	3.33	37.62
Letrozole	23.08	20.00	52.38	66.67	17.14	59.52
Gefitinib	28.57	13.33	28.57	20.00	23.33	24.29
Bicalutamide	47.62	40.00	23.81	20.00	43.81	21.90
Hydroxycarbamide	0.00	0.00	38.10	40.00	0.00	39.05
Vinorelbine	7.69	0.00	42.86	53.33	2.38	48.10
Tacrolimus	15.38	13.33	28.57	20.00	11.43	24.29
Ondansetron	0.00	0.00	23.81	20.00	0.00	21.90
**Mean Percent**	13.70	6.67	38.84	42.92	7.57	40.88

**FIGURE 1:**
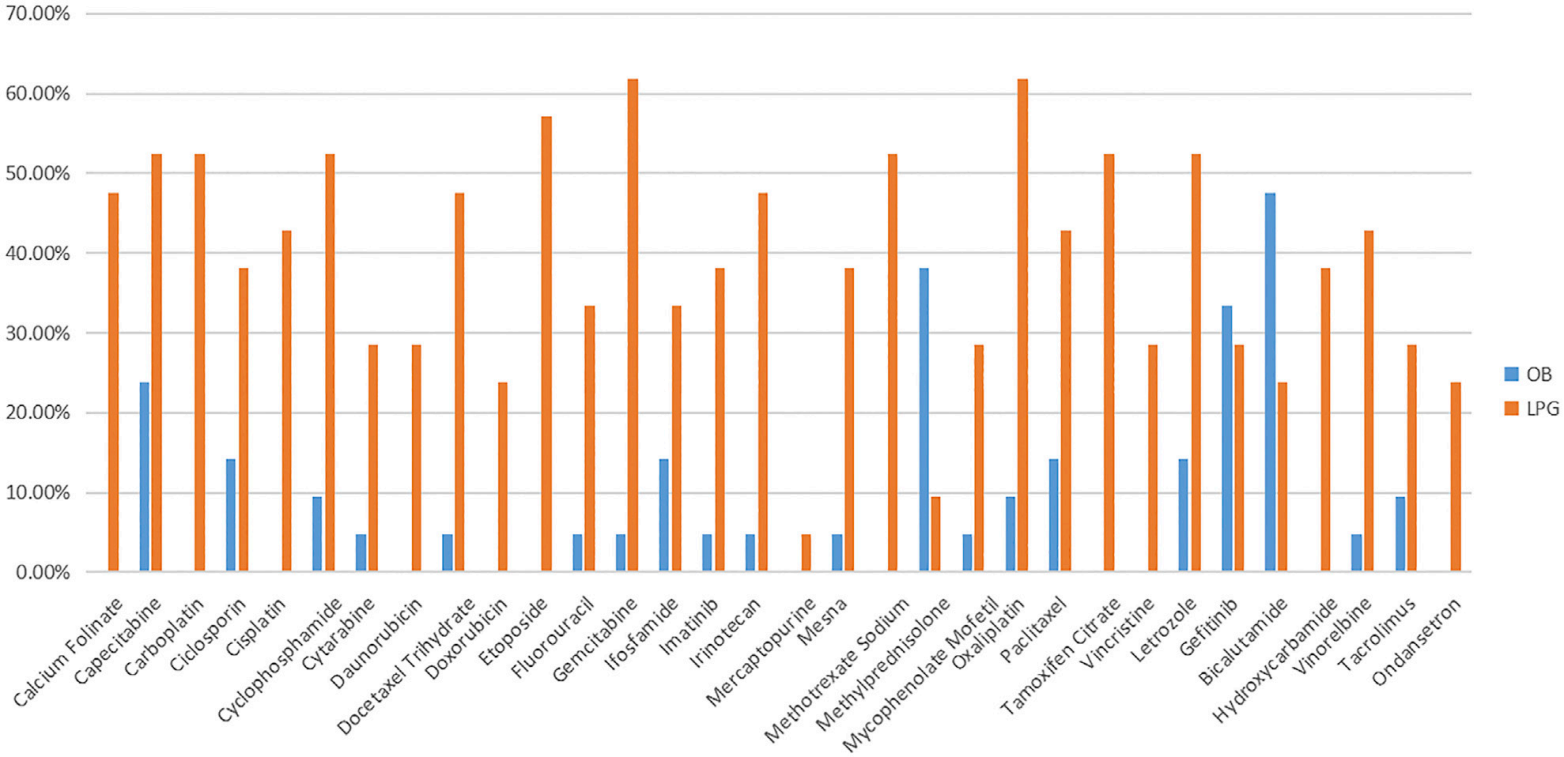
The availability of LPG and OB medicines in tertiary hospital.

**FIGURE 2:**
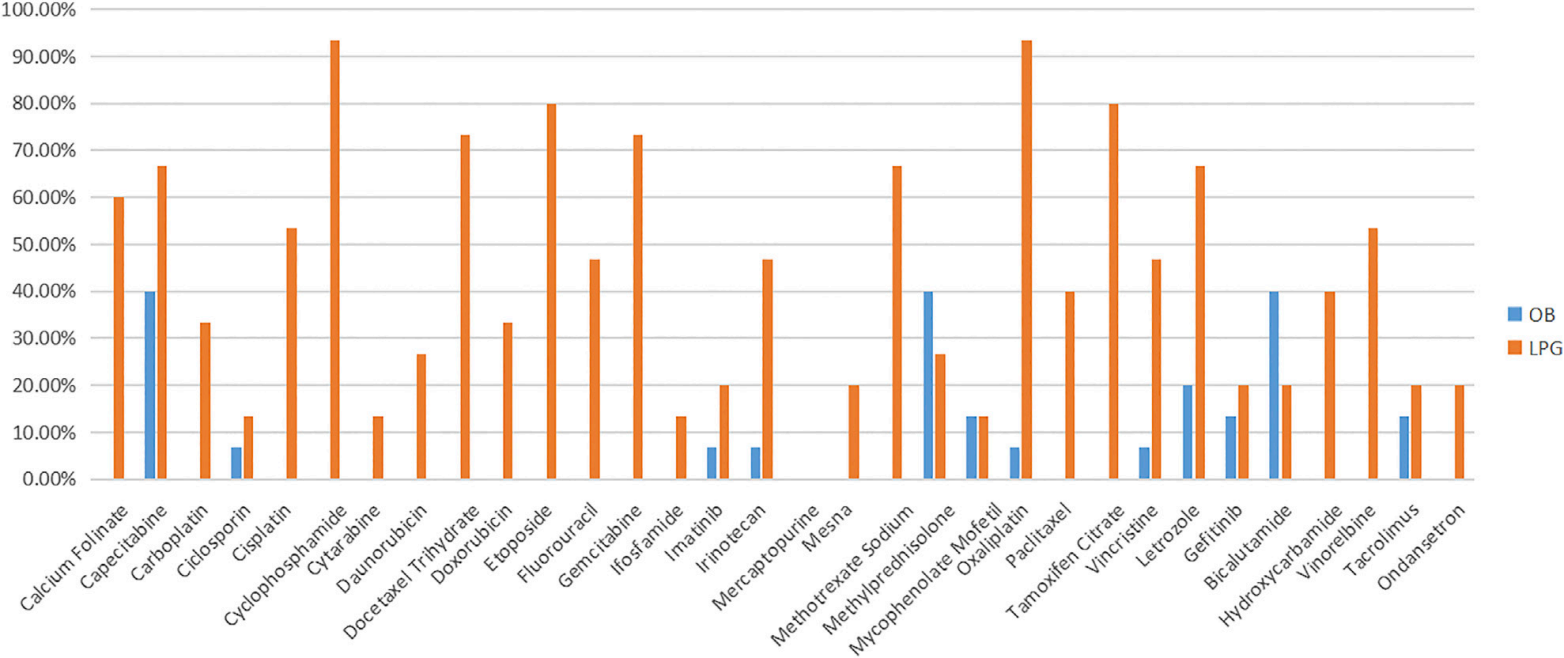
The availability of LPG and OB medicines in secondary hospital.

For LPGs, overall, the mean availability of surveyed medicines was 40.88%, and the mean availability of LPGs was 38.84% in tertiary hospitals, and 42.92% in secondary hospitals (Table 2). Besides, Oxaliplatin and Gemcitabine were maximal for 61.90% in tertiary hospitals (Figure 1). Two medicines with the highest availability in the secondary hospitals were Cyclophosphamide (93.33%) and Oxaliplatin (93.33%). Nevertheless, most LPGs were available in secondary hospitals (Figure 2). In general, the availability of the selected medicines in tertiary hospitals was higher than that in secondary hospitals.

Table 3 illustrates the median price patients paid and MPR in tertiary and secondary hospitals for 32 anticancer medicines in Hubei Province. The results showed that the overall price of medicines varied from RMB0.49 to RMB2290.12. In total, the median patient prices of 4 OBs were over double the IRP. The median patient prices of OB for Oxaliplatin (RMB2290.12) ranked first. Especially, its MPR was 12.73 times higher than the international reference price (IRP)from MSH. Furthermore, the MPR of 3 OBs were **more than double** that of IRP in tertiary hospital: Cytarabine (MPR = 17.15), Ifosfamide (MPR = 2.30), Paclitaxel (MPR = 2.81), respectively. The MPR value of sample medicines in **secondary hospitals** were less than 2, except Letrozole (MPR = 3.26). Additionally, the median patient prices of most LPGs were in line with IRP standards.

**TABLE 3:** The median patient price and MPR of anticancer medicines in tertiary and secondary hospitals in Hubei Province.

Medicine name	Medicine type	Tertiary hospital (*n* = 13)		Secondary hospital (*n* = 15)	
		Median price	MPR	Median price	MPR
Calcium Folinate	OB	NA	NA	NA	NA
	LPG	16.18	1.00	16.18	1.00
Capecitabine	OB	30.72	1.00	30.72	1.00
	LPG	8.16	0.27	11.14	0.36
Carboplatin	OB	NA	NA	NA	NA
	LPG	53.90	1.00	53.90	1.00
Ciclosporin	OB	9.15	1.00	NA	NA
	LPG	5.18	0.57	5.36	0.59
Cisplatin	OB	NA	NA	NA	NA
	LPG	7.57	1.04	7.30	1.00
Cyclophosphamide	OB	NA	NA	NA	NA
	LPG	25.20	1.00	25.20	1.00
Cytarabine	OB	140.49	17.15	NA	NA
	LPG	8.19	1.00	8.09	0.99
Daunorubicin	OB	NA	NA	NA	NA
	LPG	27.15	1.00	27.15	1.00
Docetaxel	OB	NA	NA	NA	NA
	LPG	204.84	0.66	123.00	0.40
Doxorubicin	OB	NA	NA	NA	NA
	LPG	23.28	1.00	23.28	1.00
Etoposide	OB	NA	NA	NA	NA
	LPG	7.79	0.78	9.28	0.93
Fluorouracil	OB	NA	NA	NA	NA
	LPG	9.80	1.98	5.10	1.03
Gemcitabine	OB	NA	NA	NA	NA
	LPG	150.99	1.00	79.75	0.53
Ifosfamide	OB	107.88	2.30	NA	NA
	LPG	47.00	1.00	42.21	0.90
Imatinib	OB	NA	NA	NA	NA
	LPG	14.05	0.70	14.05	0.70
Irinotecan	OB	385.87	1.66	NA	NA
	LPG	252.44	1.08	252.44	1.08
Mercaptopurine	OB	NA	NA	NA	NA
	LPG	0.62	0.26	NA	NA
Mesna	OB	NA	NA	NA	NA
	LPG	8.80	1.00	8.80	1.00
Methotrexate Sodium	OB	NA	NA	NA	NA
	LPG	19.60	1.00	19.60	1.16
Methylprednisolone	OB	123.96	1.00	125.83	1.02
	LPG	165.72	1.34	169.10	1.36
Mycophenolate Mofetil	OB	14.85	1.85	14.87	1.85
	LPG	8.04	1.00	8.31	1.03
Oxaliplatin	OB	2,290.12	12.73	NA	NA
	LPG	179.88	1.00	73.10	0.41
Paclitaxel	OB	94.19	2.81	NA	NA
	LPG	33.55	1.00	15.99	0.48
Tamoxifen Citrate	OB	NA	1.09	NA	NA
	LPG	0.58	NA	0.56	1.06
Vincristine	OB	NA	NA	NA	NA
	LPG	43.47	3.25	9.49	1.00
Letrozole	OB	37.55	1.00	37.73	3.26
	LPG	10.51	1.00	11.56	1.00
Gefitinib	OB	228	0.69	231.90	1.02
	LPG	158.4	1.32	158.50	0.70
Bicalutamide	OB	40.83	0.90	40.83	1.32
	LPG	27.88	NA	40.83	1.32
Hydroxycarbamide	OB	NA	1.00	NA	NA
	LPG	0.49	NA	0.49	1.00
Vinorelbine	OB	NA	NA	NA	NA
	LPG	134.82	1.00	134.82	1.00
Tacrolimus	OB	23.11	1.98	23.21	1.99
	LPG	10.51	0.90	16.32	1.40
Ondansetron	OB	NA	NA	NA	NA
	LPG	13.89	1.16	22.24	1.86

a The unit of measurement of Median Price is RMB.

**FIGURE 3:**
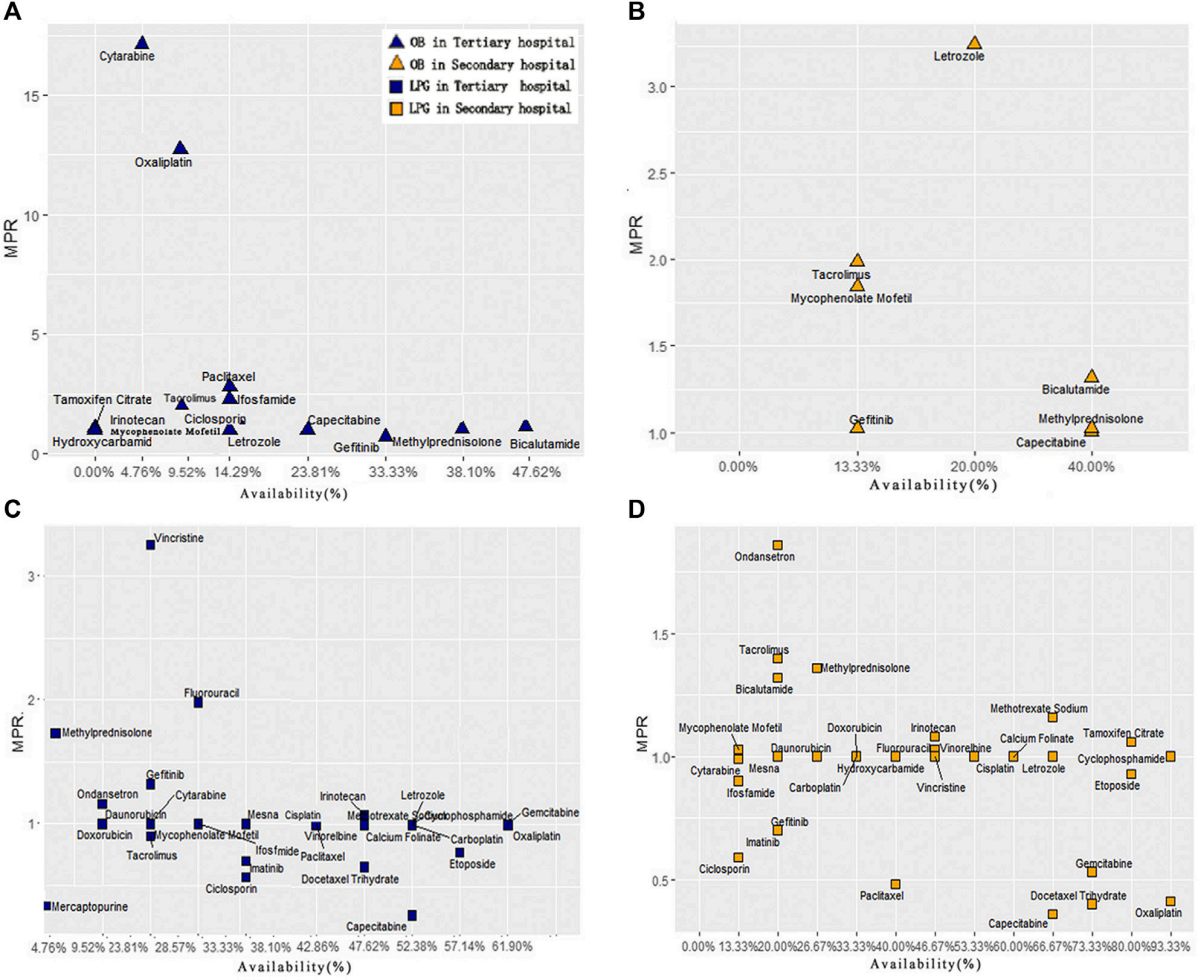
Comprehensive analysis of anticancer medicine availability and MPR in Hubei Province. **(A)** Scatter plot of OBs availability and MPR in tertiary hospitals. **(B)** Scatter plot of OBs availability and MPR in secondary hospitals. **(C)** Scatter plot of LPGs availability and MPR in tertiary hospitals. **(D)** Scatter plot of LPGs availability and MPR in secondary hospitals.

In order to compare the difference of drug availability and MPR value between secondary and tertiary hospitals, we made 4 scatter plots of the availability and MPR value of 32 anticancer drugs (Figure 3). In tertiary hospitals, for OBs, two points located in the upper left area of the graph, which were cytarabine and oxaliplatin, whose MPR was more than 10 times. However, their availability was quite low. The medicines with higher availability of OBs were gefitinib, Bicalutamide and Methylprednisolone with low MPR. For LPGs, Vincristine had the highest MPR value (3.25), and its availability was low. Eight medicines had fairly high availability with acceptable MPR value (MPR of 1 or less is interpreted as efficient procurement in the public sector) (Mendis et al., 2007; Kasonde et al., 2019).

In secondary hospitals, for OBs, only seven drugs with MPR value could be obtained. Figure 3 indicates Letrozole with high availability and MPR. Only Capecitabine’s MPR is acceptable. For LPGs, 11 medicines were highly available. Moreover, 21 drugs with MPR value were included. The result also showed that these eight medicines: Irinotecan, Methotrexate Sodium, Methylprednisolone, Mycophenolate Mofetil, Tamoxifen Citrate, Bicalutamide, Tacrolimus, Ondansetron were out of the acceptable range (MPR of 1 or less).

Table 4 shows the affordability of the anticancer medicines based on the duration of treatment. Overall, the mean affordability of the studied medicines was 2.89. For 31 LPGs, the mean affordability value was 1.79, which was within normal range. However, for 14 OBs, the mean value of affordability was 5.29. Further, irinotecan (13.89) and oxaliplatin (11.91) induced large payment burden, as the cost of 30 days dosage was higher than 10 times of 20% household minimum monthly income. In the surveyed hospitals, the affordability of 50% (7/14) of the OBs exceeded the mean affordability value (5.29), and 22.58% (7/31) of LPGs was over the average affordability.

**TABLE 4:** Affordability of anticancer medicines based on 20% of the minimum household monthly income in Hubei Province.

Medicine name	Medicine type	Dosage	Strength	Total course of treatment	Median patient price	Affordability
Calcium Folinate	OB	VIAL	100 mg/10 ml	NA	NA	0.00
	LPG	VIAL	100 mg/10 ml	10	16.18	0.32
Capecitabine	OB	TAB-CAP	500 mg	105	30.72	6.45
	LPG	TAB-CAP	500 mg	105	8.16	1.71
Carboplatin	OB	VIAL	100 mg/10 ml	6	NA	0.00
	LPG	VIAL	100 mg/10 ml	6	53.90	0.65
Ciclosporin	OB	TAB-CAP	25 mg	30	9.15	0.55
	LPG	TAB-CAP	25 mg	30	5.18	0.31
Cisplatin	OB	VIAL	20 mg	10	NA	0.00
	LPG	VIAL	20 mg	10	7.57	0.15
Cyclophosphamide	OB	VIAL	200 mg	20	NA	0.00
	LPG	VIAL	200 mg	20	25.20	1.01
Cytarabine	OB	VIAL	100 mg	20	140.49	5.62
	LPG	VIAL	100 mg	20	8.19	0.33
Daunorubicin	OB	VIAL	10 mg	6	NA	0.00
	LPG	VIAL	10 mg	6	27.15	0.33
Docetaxel	OB	VIAL	20 mg/ml	3.75	NA	0.00
	LPG	VIAL	20 mg/ml	3.75	204.84	1.54
Doxorubicin	OB	VIAL	10 mg	6	NA	0.00
	LPG	VIAL	10 mg	6	23.28	0.28
Etoposide	OB	VIAL	20 mg/ml	30	NA	0.00
	LPG	VIAL	20 mg/ml	30	7.79	0.47
Fluorouracil	OB	VIAL	0.25 g/10ml	300	NA	0.00
	LPG	VIAL	0.25 g/10ml	300	9.80	5.88
Gemcitabine	OB	VIAL	200 mg	15	NA	0.00
	LPG	VIAL	200 mg	15	150.99	4.53
Ifosfamide	OB	VIAL	500 mg	15	107.88	3.24
	LPG	VIAL	500 mg	15	47.00	1.41
Imatinib	OB	TAB-CAP	100 mg	112	NA	0.00
	LPG	TAB-CAP	100 mg	112	14.05	3.15
Irinotecan	OB	VIAL	40 mg/2 ml	18	385.87	13.89
	LPG	VIAL	40 mg/2 ml	18	252.44	9.09
Mercaptopurine	OB	TAB-CAP	50 mg	240	NA	0.00
	LPG	TAB-CAP	50 mg	240	0.62	0.30
Mesna	OB	VIAL	0.4 g/4 ml	10	NA	0.00
	LPG	VIAL	0.4 g/4 ml	10	8.80	0.18
Methotrexate Sodium	OB	VIAL	100 mg/ml	32	NA	0.00
	LPG	VIAL	100 mg/ml	32	9.80	0.63
Methylprednisolone	OB	VIAL	500 mg	20	123.96	4.96
	LPG	VIAL	500 mg	20	161.72	6.47
Mycophenolate Mofetil	OB	TAB-CAP	250 mg	240	14.85	7.13
	LPG	TAB-CAP	250 mg	240	8.04	3.86
Oxaliplatin	OB	VIAL	50 mg	2.6	2290.12	11.91
	LPG	VIAL	50 mg	2.6	179.88	0.94
Paclitaxel	OB	VIAL	30 mg/5 ml	30	94.19	5.65
	LPG	VIAL	30 mg/5 ml	30	33.55	2.01
Tamoxifen Citrate	OB	TAB-CAP	10 mg	30	NA	0.00
	LPG	TAB-CAP	10 mg	30	0.58	0.03
Vincristine	OB	VIAL	1 mg	4	NA	0.00
	LPG	VIAL	1 mg	4	134.82	1.08
Letrozole	OB	TAB-CAP	2.5 mg	75	23.11	3.47
	LPG	TAB-CAP	2.5 mg	75	10.51	1.58
Gefitinib	OB	TAB-CAP	250 mg	30	NA	0.00
	LPG	TAB-CAP	250 mg	30	13.89	0.83
Bicalutamide	OB	TAB-CAP	50 mg	30	134.82	8.09
	LPG	TAB-CAP	50 mg	7	23.11	0.32
Hydroxycarbamide	OB	TAB-CAP	500 mg	7	10.51	0.15
	LPG	TAB-CAP	500 mg	7	NA	0.00
Vinorelbine	OB	VIAL	10 mg/ml	7	13.89	0.19
	LPG	VIAL	10 mg/ml	7	134.82	1.89
Tacrolimus	OB	TAB-CAP	1 mg	6	23.11	2.77
	LPG	TAB-CAP	1 mg	6	10.51	0.13
Ondansetron	OB	TAB-CAP	4 mg	80	NA	0.00
	LPG	TAB-CAP	4 mg	80	13.89	2.22
Mean affordability	OB	5.29	LPG	1.79	Total	2.89

a Total course of treatment is calculated as 30 days' dose of anticancer medicines.

**FIGURE 4:**
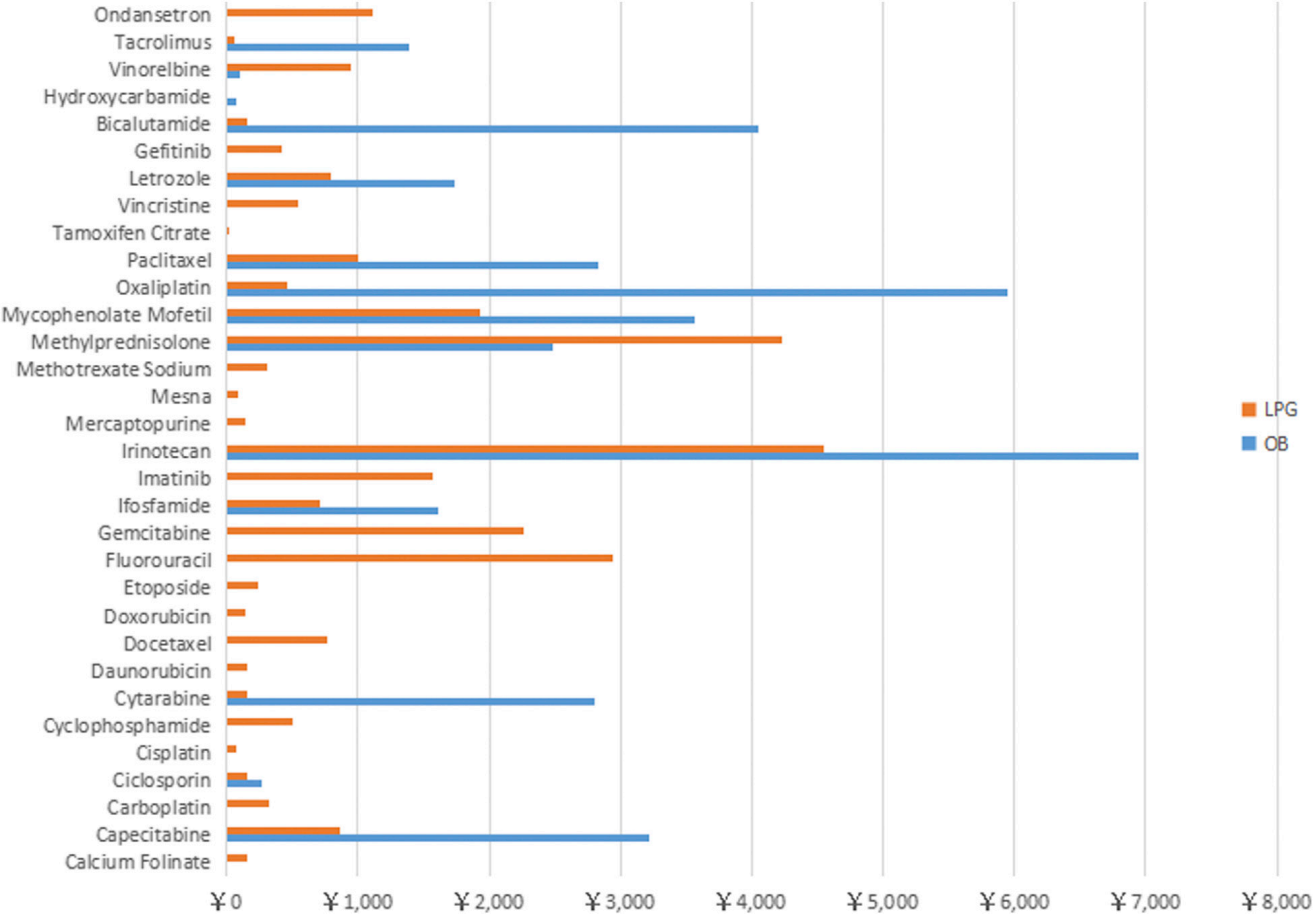
Total treatment cost of 32 anti-neoplastic for Originator Brand and Lowest Prices Generic expressed in RMB across Hubei Province.

Figure 4 shows that the monthly medicine expenditure of 3 OBs (Irinotecan, Bicalutamide, Oxaliplatin) exceeded 4000 RMB, and the cost of five medicines (Paclitaxel, Methylprednisolone, Mycophenolate Mofetil, Cytarabine and Capecitabine) were within 2000–4000 RMB. For LPGs, the 30 days treatment expenditure of four medicines (Irinotecan, Methylprednisolone, Gemcitabine, Fluorouracil) were over 2000 RMB, and the cost of most of the rest medicines were around 1000 RMB.

## Discussion

### Availability of Surveyed Medicines

The study evaluated the availability, price and affordability of 32 anticancer medicines in Hubei Province by using the WHO and HAI standard methodology. After investigating 13 tertiary hospitals and 15 secondary hospitals’ essential drugs, overall, we found that 7.57% of OB and 40.88% of LPGs were available in Hubei Province, China. The findings are similar with prior surveys in developing countries (Khuluza and Haefele-Abah, 2019; Saeed et al., 2019; Lambojon et al., 2020). Amna Saeed found that the overall availability of surveyed medicines in public sector was 6.8% only for OBs and 35.3% for the LPGs in Pakistan. In Philippines, the mean availability of surveyed medicines in the public sector was 1.3% for OBs and 25.0% for LPGs (Rui et al., 2019). They also found that most of the OB medicines were produced by large international manufacturers. Due to the lack of effective external supervision and approval for the OB medicines, price leading and brand premiums led to the relatively higher price.

Compared with secondary hospitals, the availability of surveyed medicines were higher in tertiary hospitals. Since radiotherapy and chemotherapy of tumor are very professional and expensive, they are mostly concentrated in large specialized hospitals or departments of tertiary hospitals, which contributed to the higher availability (Rui et al., 2019).

In general, the price of the OBs was high because of its core patent right of drugs, which endowed the original drug enterprises with a monopoly position (Babar et al., 2019; Zhu et al., 2019). However, LPG can be used as a competitive product to make up for its lack of accessibility (Abdel Rida et al., 2019). Especially in low and middle-income countries, hospitals will take into account the actual purchasing power of patients, and appropriately reduce the inventory proportion of OBs in hospitals (Dorj et al., 2018; Guan et al., 2018). The findings of our study showed that the availability of some medicines was relatively high, including Gemcitabine, Etoposide, Oxaliplatin, Cyclophosphamide, Tamoxifen, the underlying reason may be that these medicines could achieve better survival time in the treatment of advanced cancer and adverse reactions can be tolerated (Juxiang et al., 2015; Teng et al., 2018). Other reasons maybe they were included early in the list of national essential medicines, and widely used in the treatment of some high incidence and mortality cancers, such as lung cancer, gastric cancer, colon cancer in China (Yahong, 2019).

### Price Comparison

As presented in Figure 3, most of the LPGs for anticancer could be acquired with acceptable price. Meanwhile, for OBs, the MPR value of **Cytarabine, Oxaliplatin and Paclitaxel** were much higher than the international reference price from MSH. Compared with LPGs, the relatively retail prices for OBs were much higher. This may be explained by the large patient’s rigid demand for the above three categories of medicines. In addition, the particularity of pharmaceutical production technical and core-patient barriers made it difficult for many domestic generic drugs to reach the ideal level and curative effect of the originator brand drugs (Cameron et al., 2009).

The government has been very concerned about the field of medicine prices. In recent years, it has also issued relevant policies to curb the disordered competition in pharmaceutical market. Launching the new direction of “purchasing with quantity, linking quantity with price and combining bidding and purchasing” has pushed the price of national essential drugs back to a reasonable level (Mao et al., 2019; Dong et al., 2020). Especially, after the conformity evaluation, generic drugs gradually occupy the share of originator brand drugs and expand the OBs’ substitution in favour of lowering public health expenditure in hospitals (Faruqui et al., 2019). Diversified and fair competitions between OBs and LPGs may bring more preferential benefits to patients.

### Affordability

For the patients from low-income families in Hubei Province, they are still unable to afford course of treatment. Economic burden of disease becomes the major barrier that affects their access to effective targeted anticancer medicines (Mengyuan et al., 2017). In this study, the measurement affordability based on the total cost of treatment and the estimation of household income. The top five medicines with poor affordability were: *Irinotecan, Oxaliplatin, Bicalutamide and Mycophenolate Mofetil, Methylprednisolone*, which were more than 6 times of the 20% minimal monthly household income. The heavy payment burden may be caused by different dosages of 30 days treatment course and the unit price of each medicine. Meanwhile, in Mexico, most cancer medicines are also unaffordable for patients, the median affordability of patented medicines was 30.17 days’ wages needed to buy 1 day of one medicine’s supply (Moye-Holz et al., 2020). Hence, our government should establish special funds to support low-income household, and improve the efficiency of national medical insurance (Sarwar et al., 2018). Potential strategies worth consideration include: simplifing the approval process of new drugs at home and abroad, improving their access to the market, reducing the innovation cost, and attracking more high-quality anticancer generic drugs to enter the market (Eden et al., 2019). Importantly, in terms of the bidding and procurement process, National Health-care Security Administration negotiates with pharmaceutical manufacturers for reducing the OBs price and ensuring that the patients can afford the essential anticancer medicines (Jiao et al., 2018).

### Limitations

In this survey, private hospitals and pharmacies were not included in the survey because only few designated pharmacies were licensed to operate special drugs (such as anti-cancer drugs) in China. Hence, the use of target anticancer drugs in designated pharmacies is of particular concern in subsequent studies. Second, during data collection, some “out-of-stock” medicines were not included in the calculation. Thus, the findings may not be generalizable to the whole availability of anticancer medicines. Lastly, for the convenience in comparing the affordability indicators, but lack of accounting for detailed cancer treatment protocols, all medicines charges were calculated according to the course of 30 days. In fact, the patient’s cancer type and risk stratum are different, which could lead to some biases in the accuracy of affordability.

## Conclusion

Compared with secondary hospitals, it is obvious that the availability of essential anticancer medicines was found to be much higher in tertiary hospitals. Overall, LPGs was prior to OBs in terms of availability. “Drug addition cost” is a policy adopted by the government to compensate hospital income in a specific period of time. However, with the implementation of the “Zero Mark-up of Drugs” policy from 2009, for getting rid of compensation effect of drug income on public hospitals, the price of anticancer medicines returned to a reasonable level (Mao et al., 2019), apart from some special Originator Brand medicines’ price were still much higher than the international reference price. This study also identified several types of drugs that patients could hardly afford during the treatment. Policymakers should pay more attention to the possibility of poverty caused by expenditure on major cancer diseases. More actions should be adopted to regulate and restrict the pharmaceutical industry, drug circulation, patients and doctors, and improve the availability of essential anticancer medicines in Hubei Province.

## Data Availability

The original contributions presented in the study are included in the article/Supplementary Material, further inquiries can be directed to the corresponding author.

## Abbreviations

HAI, Health Action International; WHO, World Health Organization; EML, Essential Medicine List; OB, Originator Brand; LPG, Lowest Priced Generic; NEML, National Essential Medicine List.

## References

[B1] Abdel Rida N. Mohamed Ibrahim M. I. Babar Z. U. D. (2019). Relationship between Pharmaceutical Pricing Strategies with price, Availability, and Affordability of Cardiovascular Disease Medicines: Surveys in Qatar and Lebanon. BMC Health Serv. Res. 19 (1), 973. 10.1186/s12913-019-4828-0 31852546 PMC6921405

[B2] Babar Z. U. Ramzan S. El-Dahiyat F. Tachmazidis I. Adebisi A. Hasan S. S. (2019). The Availability, Pricing, and Affordability of Essential Diabetes Medicines in 17 Low-, Middle-, and High-Income Countries. Front. Pharmacol. 10, 1375. 10.3389/fphar.2019.01375 31824316 PMC6880243

[B3] Beran D. Ewen M. Lipska K. Hirsch I. B. Yudkin J. S. (2018). Availability and Affordability of Essential Medicines: Implications for Global Diabetes Treatment. Curr. Diab Rep. 18 (8), 48. 10.1007/s11892-018-1019-z 29907884

[B4] Cameron A. Ewen M. Ross-Degnan D. Ball D. Laing R. (2009). Medicine Prices, Availability, and Affordability in 36 Developing and Middle-Income Countries: a Secondary Analysis. Lancet 373 (9659), 240–249. 10.1016/S0140-6736(08)61762-6 19042012

[B5] China YearBooks (2019). Statistical Yearbook of Hubei Province in 2019. Wuhan city: Hubei Provincial Bureau of Statistics. available at: http://tjj.hubei.gov.cn/tjsj/sjkscx/tjnj/qstjnj/ .

[B6] Chinese, Government, Chinese, Government. Notice of the State Medical Security Administration on Bringing 17 Anticancer Drugs into Category B of the National Catalogue of Drugs for Basic Medical Insurance, Industrial Injury Insurance and Maternity Insurance ,2018.

[B7] Dong Z. Tao Q. Sun G. (2020). Survey and Analysis of the Availability and Affordability of Essential Drugs in Hefei Based on WHO/HAI Standard Survey Methods. BMC Public Health 20 (1), 1405. 10.1186/s12889-020-09477-9 32933517 PMC7493966

[B8] Dorj G. Sunderland B. Sanjjav T. Dorj G. Gendenragchaa B. (2018). Availability, Affordability and Costs of Pediatric Medicines in Mongolia. BMC Pediatr. 18 (1), 149. 10.1186/s12887-018-1123-x 29720129 PMC5932849

[B9] Eden T. Burns E. Freccero P. Renner L. Paintsil V. Dolendo M. (2019). Are Essential Medicines Available, Reliable and Affordable in Low-Middle Income Countries?. J. Cancer Pol. 19, 100180. 10.1016/j.jcpo.2018.12.001

[B10] Erratum (2020). Erratum: Global Cancer Statistics 2018: GLOBOCAN Estimates of Incidence and Mortality Worldwide for 36 Cancers in 185 Countries. CA Cancer J. Clin. 70 (4), 313. 10.3322/caac.21609 32767693

[B11] Faruqui N. Martiniuk A. Sharma A. Sharma C. Rathore B. Arora R. S. (2019). Evaluating Access to Essential Medicines for Treating Childhood Cancers: a Medicines Availability, price and Affordability Study in New Delhi, India. BMJ Glob. Health 4 (2), e001379. 10.1136/bmjgh-2018-001379 PMC6509613 31139456

[B12] Guan X. Hu H. Man C. Shi L. (2018). A Survey of Availability, price and Affordability of Essential Medicines from 2011 to 2016 in Chinese Secondary and Tertiary Hospitals. Int. J. Equity Health 17 (1), 158. 10.1186/s12939-018-0870-5 30340587 PMC6194621

[B13] Hasan S. S. Kow C. S. Dawoud D. Mohamed O. Baines D. Babar Z. U. (2019). Pharmaceutical Policy Reforms to Regulate Drug Prices in the Asia Pacific Region: The Case of Australia, China, India, Malaysia, New Zealand, and South Korea. Value Health Reg. Issues 18, 18–23. 10.1016/j.vhri.2018.08.007 30414506

[B14] Jiao Y. Yangzheng L. Lin Y. (2018). Evaluation of the Bid-Winning Effect of Centralized Procurement of Medicines in Public Hospitals in 6 central and Southern Provinces or Districts. China Pharm. 29 (15), 2017–2020.

[B15] Juxiang C. Hongxuan Z. Shao M. L. Liu M. Lianke L. (2015). Comparison between Gemcitabine Plus Oxaliplatin or S-1 and Singleagent Gemcitabine in the Treatment of Advanced Pancreatic Cancer. Anhui Med. Pharm. J. 19 (2), 359–362.

[B16] Kaiyue L. Hui L. Qian J. (2021). Overview and Analysis of National Medical Insurance Negotiation Drugs in Past Years -- Special Topic of Anti-tumor Drugs. Oncol. Pharm. 11 (02), 229–235.

[B17] Kasonde L. Tordrup D. Naheed A. Zeng W. Ahmed S. Babar Z. U. (2019). Evaluating Medicine Prices, Availability and Affordability in Bangladesh Using World Health Organisation and Health Action International Methodology. BMC Health Serv. Res. 19 (1), 383. 10.1186/s12913-019-4221-z 31196078 PMC6567665

[B18] Khanal S. Veerman L. Ewen M. Nissen L. Hollingworth S. (2019). Availability, Price, and Affordability of Essential Medicines to Manage Noncommunicable Diseases: A National Survey from Nepal. Inquiry 56, 46958019887572. 10.1177/0046958019887572 31823665 PMC6906349

[B19] Khatib R. McKee M. Shannon H. Chow C. Rangarajan S. Teo K. (2015). Availability and Affordability of Cardiovascular Disease Medicines and Their Effect on Use in High-Income, Middle-Income, and Low-Income Countries: an Analysis of the PURE Study Data. Lancet 387 (61), 61–69. 10.1016/S0140-6736(15)00469-9 26498706

[B20] Khuluza F. Haefele-Abah C. (2019). The Availability, Prices and Affordability of Essential Medicines in Malawi: A Cross-Sectional Study. PLoS One 14 (2), e0212125. 10.1371/journal.pone.0212125 30753219 PMC6372227

[B21] Khuluza F. Heide L. (2017). Availability and Affordability of Antimalarial and Antibiotic Medicines in Malawi. PLoS One 12 (4), e0175399. 10.1371/journal.pone.0175399 28419126 PMC5395150

[B22] Kunhe L. Wenjun Z. Jianru L. (2017). Analysis of Economic burden of Cancer Patients in Hubei Province. J. Med. Soc. 30 (8), 1–3.

[B23] Lambojon K. Chang J. Saeed A. Hayat K. Li P. Jiang M. (2020). Prices, Availability and Affordability of Medicines with Value-Added Tax Exemption: A Cross-Sectional Survey in the Philippines. Int. J. Environ. Res. Public Health 17 (14). 10.3390/ijerph17145242 PMC7400398 32708060

[B24] Liang Z. Qiuyun Z. Xuemeng Z. (2020). Investigation and Study on the Accessibility of Antibiotics in Essential Medicine List in Medical Institutions. China Pharm. 31 (11), 1281–1287. 10.6039/j.issn.1001-0408.2020.11.01

[B25] Mao W. Huang Y. Chen W. (2019). An Analysis on Rational Use and Affordability of Medicine after the Implementation of National Essential Medicines Policy and Zero Mark-Up Policy in Hangzhou, China. PLoS One 14 (3), e0213638. 10.1371/journal.pone.0213638 30870490 PMC6417690

[B26] Mendis S. Fukino K. Cameron A. Laing R. Filipe A. Khatib O. (2007). The Availability and Affordability of Selected Essential Medicines for Chronic Diseases in Six Low- and Middle-Income Countries. Bull. World Health Organ. 85 (4), 279–288. 10.2471/blt.06.033647 17546309 PMC2636320

[B27] Mengyuan T. Dan C. Yuxiao Z. Xiao Y. Xin F. Jianglin H. (2017). Evaluation of the Affordability of Three Antitumor Targeted Drugs: a Case Study of Hubei Province. Chin. Pharm. 28 (20), 2746–2749.

[B28] Moye-Holz D. Ewen M. Dreser A. Bautista-Arredondo S. Soria-Saucedo R. van Dijk J. P. (2020). Availability, Prices, and Affordability of Selected Essential Cancer Medicines in a Middle-Income Country - the Case of Mexico. BMC Health Serv. Res. 20 (1), 424. 10.1186/s12913-020-05167-9 32410676 PMC7222474

[B29] Rui S. Zhimei X. Yue L. (2019). A Study on the Availability and Affordability of Anti-tumor Drugs in Public Hospitals--Based on the Data Analysis of the Typical Survey of Public Hospitals in Jiangsu Province. Price Theor. Pract. (5), 39–42.

[B30] Saeed A. Saeed H. Saleem Z. Fang Y. Babar Z. U. (2019). Evaluation of Prices, Availability and Affordability of Essential Medicines in Lahore Division, Pakistan: A Cross-Sectional Survey Using WHO/HAI Methodology. PLoS One 14 (4), e0216122. 10.1371/journal.pone.0216122 31022276 PMC6483245

[B31] Sarwar M. R. Iftikhar S. Saqib A. (2018). Availability of Anticancer Medicines in Public and Private Sectors, and Their Affordability by Low, Middle and High-Income Class Patients in Pakistan. BMC Cancer 18 (1), 14. 10.1186/s12885-017-3980-3 29298681 PMC5753448

[B32] Shaoqing L. (2019). The Status Quo and Dilemma, hope and Way Out of Cancer–Based on the analysis of the global cancer incidence, prevention and control situation in 2017 and 2018. Med. Philos. 40 (12), 20–23.

[B33] Smith S. K. Nicolla J. Zafar S. Y. (2014). Bridging the gap between financial distress and available resources for patients with cancer: a qualitative study. J. Oncol. Pract. 10 (5), e368–72. 10.1200/JOP.2013.001342 24865219 PMC5706140

[B34] Teng J. P. Yang Z. Y. Zhu Y. M. Ni D. Zhu Z. J. Li X. Q. (2018). Gemcitabine and cisplatin for treatment of lung cancer *In Vitro* and vivo. Eur. Rev. Med. Pharmacol. Sci. 22 (12), 3819–3825. 10.26355/eurrev_201806_15266 29949158

[B35] Wu G. Gong S. Cai H. Ding Y. (2018). The availability, price and affordability of essential antibacterials in Hubei province, China. BMC Health Serv. Res. 18 (1), 1013. 10.1186/s12913-018-3835-x 30594189 PMC6310993

[B36] Yahong L. (2019). Study on Family Burden, Quality of Life and Influencing Factors of Lung Cancer Patients in a Cancer Hospital. Lanzhou City: Lan Zhou University.

[B37] Yf D. Research on Public Security Policy and Drug Accessibility Evaluation of Novel Antitumor Drugs in China. Beijing: Peking Union Medical College.

[B38] Zhu Y. Wang Y. Sun X. Li X. (2019). Availability, Price and Affordability of Anticancer Medicines: Evidence from Two Cross-Sectional Surveys in the Jiangsu Province, China. Int. J. Environ. Res. Public Health 16 (19), 3728. 10.3390/ijerph16193728 PMC6801951 31623326

